# Taeniasis: An unexpected variceal hemorrhage trigger

**DOI:** 10.1016/j.idcr.2021.e01087

**Published:** 2021-03-26

**Authors:** Emmanuel Faure, Matthieu Deckmyn

**Affiliations:** a Faculté de Médecine de Lille, Université de Lille Nord de France, Lille, France; b CHU Lille, Service des Maladies Infectieuses, Centre Hospitalier Universitaire de Lille, F 59000, Lille, France; c Service de réanimation médicale et chirurgicale, Centre Hospitaliser de Roubaix, F-59100, Roubaix, France

**Keywords:** Taeniasis, Hematemesis, Upper digestive tract

## Abstract

Here we report a fatal and uncommon case of esophageal variceal bleeding secondary to the presence of a Tapeworm in the upper digestive tract in a patient coming back from North Africa.

A 54 year-old man with a history of liver cirrhosis (Child-Pugh B8) was admitted to the intensive care unit for hemorrhagic shock due to hematemesis. The patient promptly received intravenous colloid fluids and red blood cell transfusions. Due to hemodynamic instability, immediate gastroduodenal endoscopy was performed. Acute esophageal variceal bleeding was observed. Surprisingly, we identified a segmented tapeworm extending from the esophagus through the cardia to the duodenum (Fig. 1) as well as an erosive esophagitis along its path. Since the patient had no known recent history of variceal bleeding triggers, this uncommon esophageal localization of taeniasis probably eroded the esophageal varices and promoted bleeding. Indeed, helminthic infestation [1] is a known cause for occult lower intestinal bleeding [2] in developing countries but remains a very uncommon cause of acute hematemesis. Despite rapid placement of a Blakemore tube and continuing resuscitation, the patient died of uncontrolled hemorrhage after several hours.

**Fig. 1:**
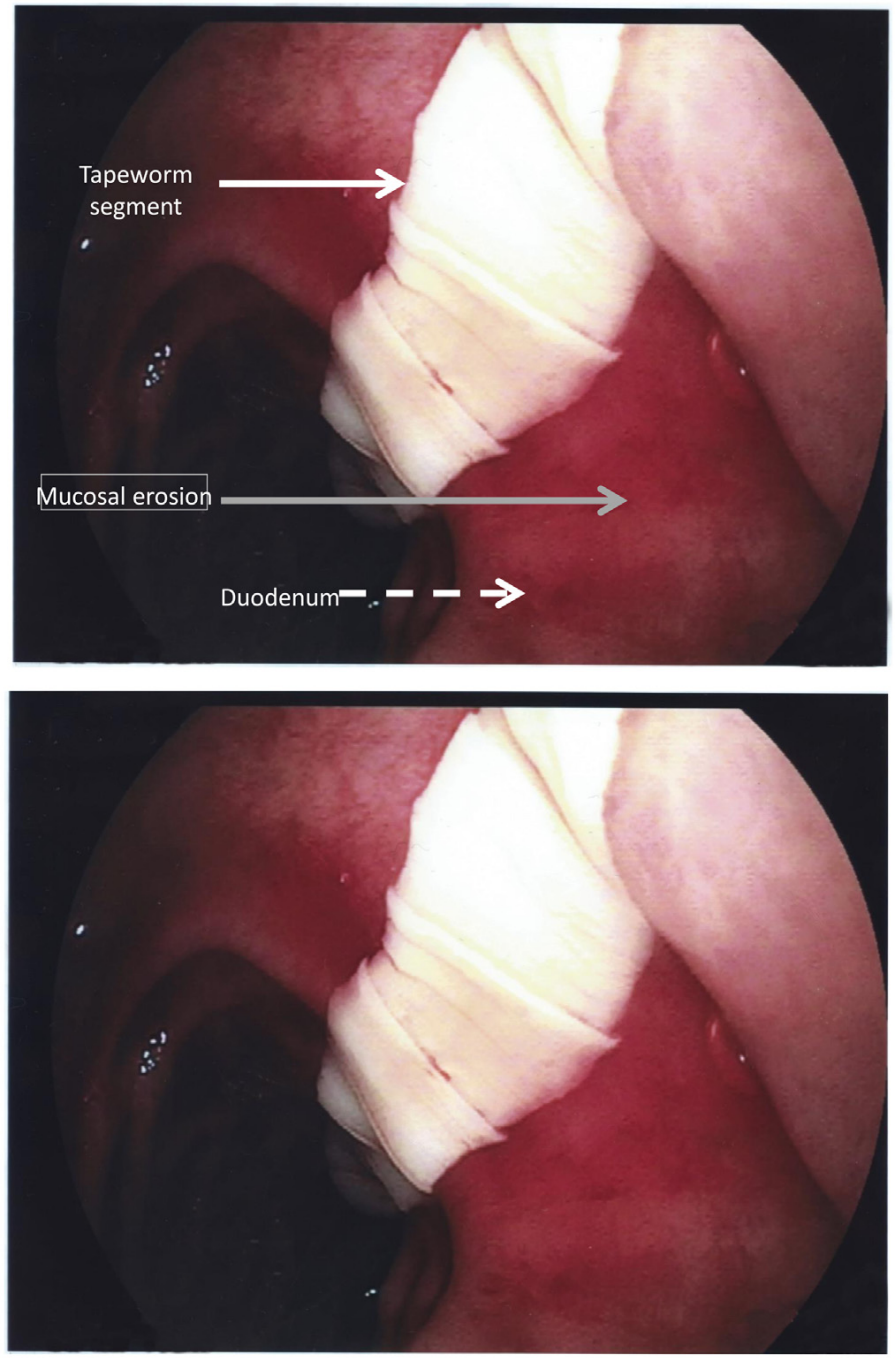
**Gastroscopy screenshot of Taeniasis on erosive duodenal mucosa.** A metameristic tapeworm compatible with Taeniasis (white solid arrow) extending from the esophagus through the cardia to the duodenum (white dashed arrow) as well as an erosive mucosa along its path (Grey Arrow).

## Declaration of Competing Interest

The authors report no declarations of interest.

## Funding source

Authors have no funding sources.

## Consent

"Written informed consent was obtained from the patient for publication of this case report and accompanying images. A copy of the written consent is available for review by the Editor-in-Chief of this journal on request”.

## Authors contribution

E.F and M.D. were physicians in charge of patient.

E.F. wrote the manuscript.

E.F. and M.D. reviewed manuscript.

## Ethical approval

Consent to publish was obtained from the patient’s relative.

## References

[1] Ng-Nguyen D., Stevenson M.A., Traub R.J. (2017). A systematic review of taeniasis, cysticercosis and trichinellosis in Vietnam. Parasit Vectors.

[2] Settesoldi A., Tozzi A., Tarantino O. (2017). Taeniasis: a possible cause of ileal bleeding. World J Clin Cases.

